# Interleukin-10 facilitates the selection of patients for systemic thrombolysis

**DOI:** 10.1186/1471-2377-13-62

**Published:** 2013-06-17

**Authors:** Manuel Rodríguez-Yáñez, Mar Castellanos, Tomás Sobrino, David Brea, Pedro Ramos-Cabrer, Salvador Pedraza, José A Castiñeiras, Joaquín Serena, Antonio Dávalos, José Castillo, Miguel Blanco

**Affiliations:** 1 Department of Neurology, Clinical Neurosciences Research Laboratory, Hospital Clínico Universitario, Universidad de Santiago de Compostela, IDIS, Santiago de Compostela, Spain; 2 Department of Neurology, Hospital Universitario Doctor Josep Trueta, Girona, Spain; 3 Department of Neurology, Hospital Universitario Germans Trias i Pujol, Universidad Autónoma de Barcelona, Barcelona, Spain; 4 Department of Radiology (Institut de Diagnostic per la Imatge [IDI]), Hospital Universitario Doctor Josep Trueta, IDIBGi, Girona, Spain; 5 Department of Neuroradiology, Hospital Clínico Universitario, Santiago de Compostela, Spain; 6 Universidad de Santiago de Compostela, IDIS, Santiago de Compostela, Spain

**Keywords:** Ischemic stroke, Thrombolysis, Interleukin-10, MRI

## Abstract

**Background:**

Clinical-Diffusion mismatch (CDM; NIHSS score ≥8 & DWI lesion volume ≤25 mL) and Perfusion-Diffusion mismatch (PDM; difference >20% between initial DWI and MTT lesion volumes) have been proposed as surrogates for ischemic brains that are at risk of infarction. However, their utility to improve the selection of patients for thrombolytic treatment remains controversial. Our aim was to identify molecular biomarkers that can be used with neuroimaging to facilitate the selection of ischemic stroke patients for systemic thrombolysis.

**Methods:**

We prospectively studied 595 patients with ischemic stroke within 12 h of the stroke onset. A total of 184 patients received thrombolytic treatment according to the SITS-MOST criteria. DWI and MTT volumes were measured at admission. The main outcome variable was good functional outcome at 3 months (modified Rankin scale <3). Serum levels of glutamate (Glu), IL-10, TNF-α, IL-6, NSE, and active MMP-9 also were determined at admission.

**Results:**

Patients treated with t-PA who presented with PDM had higher IL-10 levels at admission (p < 0.0001). In contrast, patients with CDM had higher levels of IL-10 (p < 0.0001) as well as Glu and TNF-α (all p < 0.05) and lower levels of NSE and active MMP-9 (all p < 0.0001). IL-10 ≥ 30 pg/mL predicts good functional outcome at 3 months with a specificity of 88% and a sensitibity of 86%. IL-10 levels ≥30 pg/mL independently in both patients with PDM (OR, 18.9) and CDM (OR, 7.5), after an adjustment for covariates.

**Conclusions:**

Serum levels of IL-10 facilitate the selection of ischemic stroke patients with CDM and PDM for systemic thrombolysis.

## Background

Systemic thrombolysis during the first 4.5 hours after the onset of a stroke is the gold standard for the treatment of acute ischemic stroke. However, t-PA has been linked to severe side effects, can be applied only in a restricted number of patients, and has only moderate efficacy. Further criteria need to be developed that may expand the time window for treatment. Neuroimaging methods have been developed in recent years to identify the presence of ischemic brain tissue that is still viable. The identification of this at-risk tissue would increase the therapeutic window beyond 4.5 hours and improve the selection of patients for recanalization. The perfusion-diffusion mismatch (PDM) model has been the most frequently used to identify at-risk tissue. However, in addition to technical difficulties, the cost effectiveness of PDM is limited, and recent studies have challenged its utility in selecting patients for systemic thrombolysis [1-3]. Our group has previously proposed the clinical-diffusion mismatch (CDM) as a simplified alternative method to select patients for thrombolytic therapy [4]. CDM is based on the supposition that patients with severe neurological deficits (NIHSS ≥8) but relatively small lesion volumes on diffusion weighted images (DWI volume <25 mL) are likely to have enough of an ischemic penumbra to benefit from revascularization. Subsequent studies by other groups have confirmed the utility of CDM [5-7]. However, the usefulness of CDM to improve the selection of patients for thrombolytic treatment has not been confirmed.

Moreover, previous studies by our group have demonstrated that salvageable brain, which is defined as the presence of CDM, may be predicted by a number of molecular signatures of ischemic but non-infarcted brain. Specifically, higher levels of IL-10, TNF-α and Glu as well as lower levels of NSE, IL-6 and active MMP-9 have been associated with the presence of CDM [8].

Our aim was to study whether serum levels of several biomarkers could facilitate the selection of ischemic stroke patients with CDM or PDM for systemic thrombolytic treatment with t-PA.

## Methods

### Study population and patients characteristics

We prospectively evaluated 835 consecutive patients with hemispheric ischemic stroke who were admitted to two university hospitals within the first 12 hours of their stroke onset. Patients who presented with stroke on awakening (n = 38), severe systemic disease (n = 27), dementia (n = 9), lesions located within the vertebrobasilar territory (n = 13), psychiatric disease (n = 9), chronic inflammatory disease (n = 21), or active infection (n = 11), or were enrolled in other clinical trials (n = 55), refused to participate in the study (n = 4), had technical problems with the MRI (n = 9), were lost to follow-up (n = 36) or were undergoing other recanalization procedures (n = 8) were excluded from the study. Therefore, a total of 595 patients were included in the study.

The Local Ethical Committees of the Clinical University Hospital of Santiago de Compostela and the Clinical University Hospital Doctor Josep Trueta of Girona approved the protocol. All patients or their relatives gave signed informed consent.

### Clinical variables and neuroimaging studies

All patients were treated according to the guidelines for the management of ischemic stroke from the European Stroke Organization (ESO) [9]. A total of 184 patients received thrombolytic treatment with t-PA within the first 3 hours of the stroke onset following the SITS-MOST criteria [10]. Demographic data (age, sex), and a prior history of high blood pressure, diabetes, heart disease, hyperlipidemia, alcohol consumption and smoking were recorded. At admission, a blood sample was collected to determine the blood count as well as other biochemistry and molecular markers, and diffusion-weighted (DWI) and perfusion-weighted (PWI) magnetic resonance imaging (MRI) was performed. The stroke subtype was classified according to the TOAST criteria [11] once carotid and transcranial ultrasounds and ancillary studies were completed. The National Institute of Health Stroke Scale (NIHSS) score (admission, 24 h, 48 h, 72 h and 90 ± 7 days) and the modified Rankin Scale (mRS) (90 ± 7 days) were evaluated by certified investigators. The functional outcome was categorized as good (mRS <3) or poor (mRS ≥3).

MR images were obtained at admission on a 1.5 T system (Intera, Philips, Best, The Netherlands) or a 1.5 Magneton Symphony (Siemens, Er- langen, Germany) with echoplanar capabilities of 25-mT/m gradients and 300- to 350-microsecond rise times. The MRI protocol included DWI, T2-weighted, and fluid-attenuated inversion recovery (FLAIR) imaging. The DWI sequence was performed with a b value of 1,000 and was analysed in the trace image to avoid anisotropy. The PWI lesion volume was measured using mean transient time (MTT) maps with a 4-second threshold. PDM was defined as positive when the difference between the DWI and MTT lesion volumes was >20%. Based on previous studies, we defined the presence of CDM as an NIHSS score ≥ 8 at admission and a lesion volume during the baseline DWI < 25 mL [4]. A second MRI to evaluate the DWI volume was performed 72 ± 12 hours in all patients that received thrombolytic therapy, except in 6 patients who died. We evaluated the infarct growth as the percentage difference in DWI volume between the basal MRI (DWI 0 h) and the control MRI (DWI 72 h) according to the following formula: (DWI 72 h - DWI 0 h)/DWI 0 h × 100.

A neuroradiologist who was blind to the clinical data evaluated the DWI and MTT volumes by manual segmentation method.

### Laboratory test

Blood samples, which were obtained from all patients at admission before thrombolytic therapy, were collected in chemistry test tubes, centrifuged at 3000 g for 15 minutes, and immediately frozen and stored at −80°C. Serum levels of interleukin (IL)-6 and tumour necrosis factor (TNF)-α were measured using an immunodiagnostic IMMULITE 1000 System (Diagnostic Products Corporation, California, USA). Neuron-specific enolase (NSE) serum levels were determined by electrochemiluminescence immunoassay using an analyser ELECSYS 2010 (Roche Diagnostics GmbH, Mannheim, Germany). Likewise, the serum levels of IL-10 (Bender Medsystems GmbH, Vienna, Austria) as well as the active metalloproteinase-9 (MMP-9) (GE Healthcare - Amersham, Little Chalfont Buckinghamshire, UK) were measured using commercial ELISA kits following the manufacturer instructions. Serum levels of glutamate (Glu) were determined by high performance liquid chromatography (HPLC) analysis following a previously described method [12]. The intra-assay and inter-assay coefficients of variation (CV) for each molecular marker are reported in Additional file 1: Table S1. Determinations were performed in a laboratory that was blind to the clinical and neuroimaging data.

### Statistical analysis

Results are expressed as percentages for the categorical variables and as either the mean (SD) or median [quartiles] for the continuous variables depending on whether or not, respectively, the data followed a normal distribution. Proportions were compared using the chi-square test. The Student’s t test (normal data) or the Mann–Whitney test (non-normal data) was used to compare continuous variables between 2 groups, and the ANOVA test was used to compare across more than 2 groups. Bivariate correlations were determined by the Pearson’s correlation coefficient. ROC curves were configured to establish cut-off points for molecular marker levels that optimally predicted good functional outcome.

Due to the colinearity between the molecular markers, the influence of each marker on the functional outcome at 3 months was assessed in a separate logistic regression model and adjusted for the main baseline variables that were related to good functional outcome (mRS <3) in the univariate analyses (p < 0.05). Results were expressed as adjusted odds ratios (OR) with the corresponding 95% confidence intervals (95% CI). Statistical analyses were conducted using SPSS 16.0 for Mac.

## Results

All patients had DWI-MRI performed at admission, and 237 patients (39.8%) had PWI-MRI performed at admission. CDM was found in 244 patients (41.0%) and PDM in 189 of 237 patients (79.7%).

**Figure 1:**
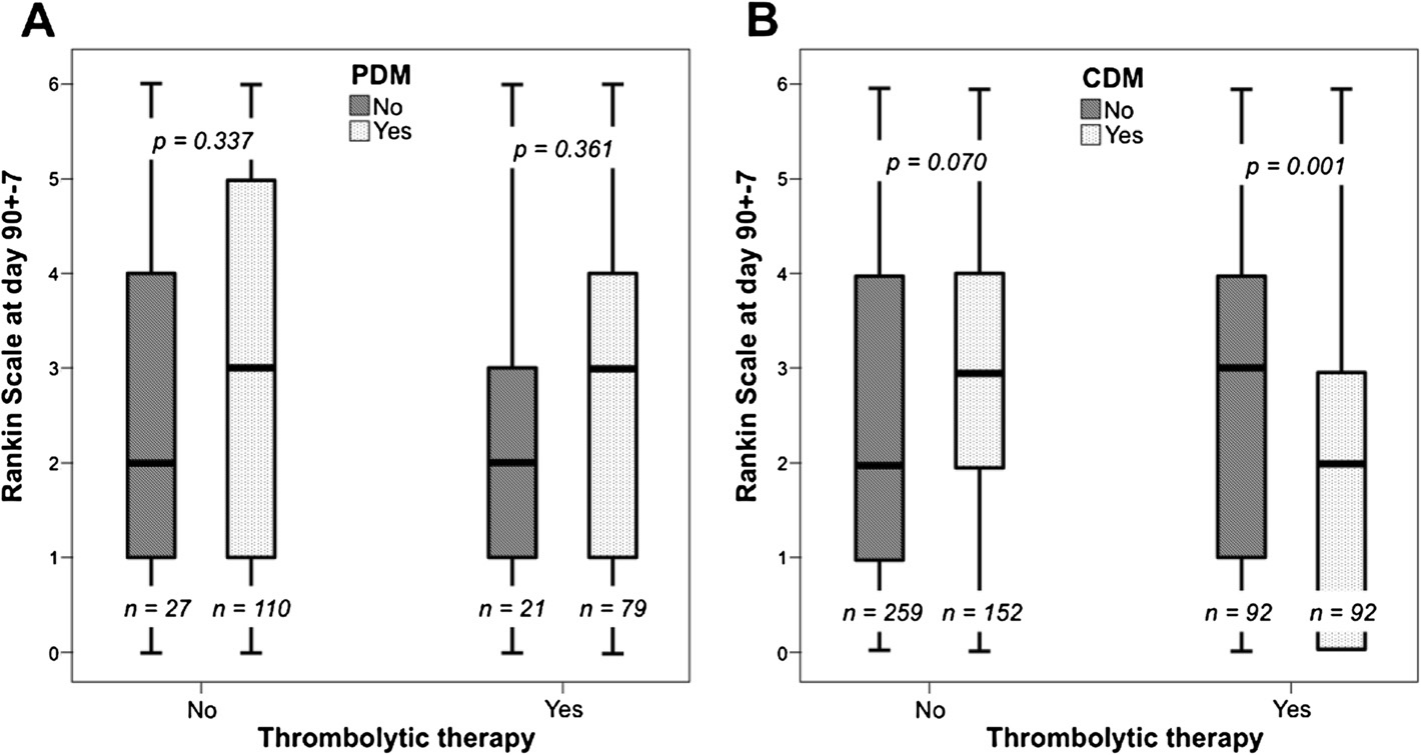
**Median [quartiles] of the modified Rankin Scale (mRS) scores in patients who either received or did not receive thrombolytic treatment.** Figure **A** shows patients with and without perfusion-diffusion mismatch (PDM) and Figure **B** shows patients with and without clinical-diffusion mismatch (CDM).

No differences in functional outcome at 3 months were found in patients with or without CDM or PDM who did not received thrombolytic therapy. However, patients with CDM who received thrombolytic treatment showed a better outcome than patients without CDM (3 [1,4] vs. 2 [0,3], p = 0.001). In contrast, a good functional outcome at 3 months was independent of PDM status (2 [1,4] vs. 3 [1,4], p = 0.361) (Figure 1).

A total of 184 patients (30.9%) received thrombolytic treatment according to the SITS-MOST criteria. Patients who presented with PDM and were treated with t-PA had higher IL-10 levels at admission. In contrast, patients with CDM had higher levels of IL-10 as well as Glu and TNF-α and lower levels of NSE and active MMP-9 (Table 1).

**Table 1:** Molecular profiles of patients with perfusion-diffusion mismatch (PDM) and clinical-diffusion mismatch (CDM) who received or did not receive thrombolytic treatment

	**PDM (n = 100)**			**CDM (n = 184)**		
	**NO (n = 21)**	**YES (n = 79)**	**p**	**NO (n = 92)**	**YES (n = 92)**	**p**
NSE (ng/mL)	22.4 ± 9.0	20.7 ± 6.1	0.449	24.6 ± 8.3	21.4 ± 5.8	0.002
IL-10 (pg/mL)	8.8 ± 7.0	22.5 ± 14.9	<0.0001	8.6 ± 8.0	30.9 ± 14.7	<0.0001
Glutamate (μM)	150.9 ± 102.2	207.7 ± 177.9	0.290	152.5 ± 91.5	226.6 ± 186.9	0.031
IL-6 (pg/mL)	9.9 ± 8.9	11.9 ± 8.6	0.250	20.9 ± 17.7	14.2 ± 7.6	0.109
TNF-α (pg/mL)	10.3 ± 6.3	11.5 ± 8.2	0.774	12.4 ± 6.6	18.4 ± 12.6	0.003
MMP-9 (ng/mL)	24.0 ± 16.6	21.0 ± 11.9	0.745	29.6 ± 18.4	20.2 ± 8.4	<0.0001

Table 2 shows the baseline characteristics of patients who received thrombolytic treatment by outcome group. A good outcome was observed in 103 patients (56.0%) and a poor outcome was observed in 81 patients (44.0%). Patients with a good outcome were younger and had lower stroke severity and smaller DWI and MTT volumes at admission compared with patients with a poor outcome. The only molecular marker that was independently associated with a good outcome in patients treated with t-PA was IL-10, which was significantly associated with a good outcome in patients with PDM (OR: 1.08, CI 95%: 1.94-17.22) as well as CDM (OR: 1.09, CI 95%: 1.04-1.13). We found that an IL-10 level ≥ 30 pg/mL predicts a good functional outcome at 3 months with a specificity of 88% and a sensibility of 86% (area under the curve, 0.952; p < 0.0001).

**Table 2:** Baseline characteristics of patients who received thrombolytic treatment by outcome groups

	**Poor outcome (n = 81)**	**Good outcome (n = 103)**	**p**
Age, years	71.4 ± 9.3	64.3 ± 13.5	<0.0001
Male gender, %	54.3	61.2	0.370
Time from stroke onset, minutes	84.7 ± 36.4	102.9 ± 83.8	0.234
TOAST			0.095
- Atherothrombotic, n (%)	16.0	13.6	
- Cardioembolic, n (%)	59.3	49.5	
- Lacunar, n (%)	0	5.8	
- Undetermined, n (%)	24.7	31.1	
History of hypertension, n (%)	59.3	50.5	0.297
History of diabetes, n (%)	12.3	9.7	0.637
History of dyslipemia, n (%)	21.0	32.0	0.099
Smoking history, n (%)	14.8	12.6	0.672
Alcohol consumption, n (%)	6.2	9.7	0.429
History of coronary disease, n (%)	11.1	12.6	0.822
History of atrial fibrillation, n (%)	35.8	25.2	0.145
Previous stroke or TIA, n (%)	14.8	8.7	0.245
Systolic blood pressure, mmHg	159.1 ± 24.4	155.6 ± 27.6	0.327
Diastolic blood pressure, mmHg	86.7 ± 16.5	83.5 ± 15.1	0.180
Body temperature, °C	36.2 ± 2.3	36.4 ± 0.4	0.218
Glucose levels, mg/dL	129.5 ± 32.2	126.3 ± 40.0	0.142
Leukocytes, 10^3^/mL	8.2 ± 2.3	8.7 ± 2.9	0.349
Platelets, 10^3^/mL	223.6 ± 64.0	247.3 ± 86.7	0.086
Fibrinogen, mg/dL	433.7 ± 114.7	412.6 ± 138.3	0.069
NIHSS at admission	18 [14, 20]	11 [7, 16]	<0.0001
DWI volume at admission, mL	56.0 ± 60.2	16.7 ± 20.2	<0.0001
MTT volume at admission, mL	143.2 ± 87.4	72.3 ± 79.9	<0.0001
Neuron-specific enolase (ng/mL)	23.8 ± 7.4	22.4 ± 7.2	0.202
Interleukin-10 (pg/mL)	12.9 ± 10.7	25.1 ± 17.9	<0.0001
Tumor necrosis factor-α (pg/mL)	13.8 ± 8.7	16.7 ± 11.5	0.057
Interleukin-6 (pg/mL)	21.1 ± 17.3	14.8 ± 9.9	0.002
Active metalloproteinase-9 (ng/mL)	27.8 ± 17.7	22.6 ± 12.1	0.026
Glutamate (μM)	212.7 ± 147.5	171.4 ± 152.6	0.065

**Figure 2:**
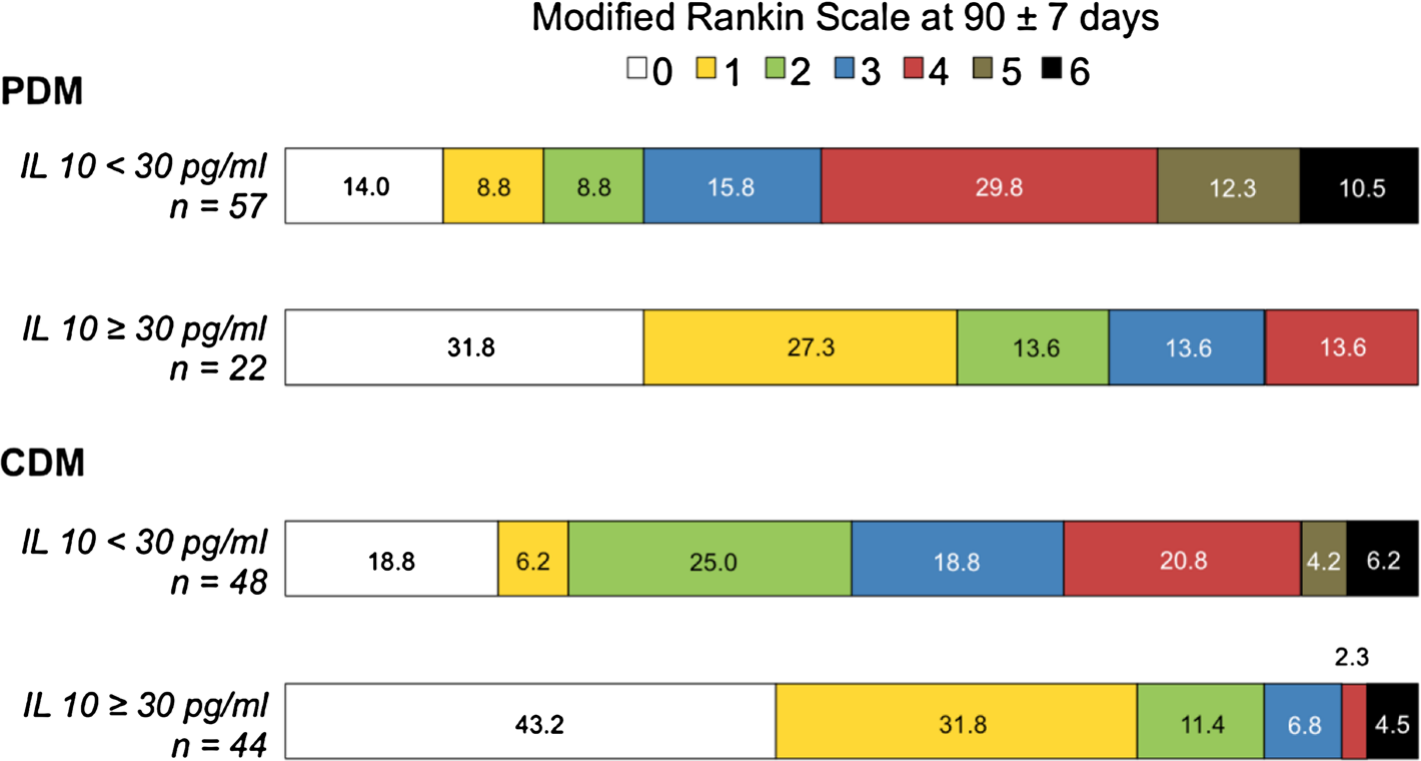
Distribution of the modified Rankin Scale (mRS) scores in thrombolytic patients with PDM and CDM categorized by IL-10 levels.

Figure 2 shows the distribution of mRS scores in the thrombolytic patients with PDM and CDM categorized by IL-10 levels. Patients with PDM who also had IL-10 levels ≥30 mg/mL had a good outcome more frequently than patients with PDM with lower IL-10 levels (72.7 vs. 31.6%, p = 0.002). Similar results were found for patients with CDM and IL-10 levels ≥30 mg/mL (86.4 vs. 50.0%, p < 0.0001). In the logistic regression model, IL-10 levels ≥30 mg/mL were independently associated with a good functional outcome at 3 months in patients with PDM (OR: 18.88, CI 95%: 3.13-53.03) and CDM (OR: 7.47, CI 95%: 2.52-22.12), after adjusting for age and NIHSS at admission (The DWI volume was not included in this model because it is a surrogate variable of CDM and PDM. However, in a logistic regression model that included DWI volume at admission, the IL-10 levels maintain independence. These data are shown in Table 3).

**Table 3:** Logistic regression model including those variables that were significant in the univariate model forcing the inclusion of DWI volume at admission (model 1) and DWI volume and NIHSS score and admission (model 2)

**Model 1**	**OR**	**CI 95%**
IL-10 levels	1.05	1.01-1.08
IL-10 ≥30 pg/mL	2.86	1.06-7.82
DWI volume	0.98	0.97-0.98
Model 2	OR	CI 95%
IL-10 levels	1.07	1.03-1.11
IL-10 ≥30 pg/mL	3.14	1.04-9.51
DWI volume	0.99	0.99-1.00
NIHSS score	0.77	0.73-0.81

In patients who were treated with t-PA, IL-10 serum levels were negatively correlated with an increase in DWI volume during the first 72 hours (Pearson’s coefficient = −0.330, p < 0.0001). This increase in DWI volume during the first 72 hours was associated with higher glutamate levels (Pearson’s coefficient = 0.336, p < 0.0001). This correlation between glutamate levels and an increase in DWI volume during the first 72 hours was higher in patients with IL-10 levels <30 mg/mL (Pearson’s coefficient = 0.613, p < 0.0001) and disappeared in patients with IL-10 levels ≥30 mg/mL (Pearson’s coefficient = 0.193, p = 0.056).

## Discussion

We have found that IL-10 levels are associated with the presence of PDM and CDM, and that IL-10 levels ≥30 pg/mL are independently associated with a good outcome in thrombolytic patients with both PDM and CDM. Therefore, IL-10 serum levels may be used in combination with PDM or CDM to improve the selection of patients for recanalization because patients with CDM or PDM and IL-10 ≥30 mg/mL have a better functional outcome at 3 months.

The value of PDM is controversial. Currently, no evidence exists to support or refute its significance in the diagnosis of ischemic stroke [2]. Several studies have found that PDM is not associated with either functional outcome or infarct growth in patients with ischemic stroke with or without tPA treatment [13,14]. CDM is a more accessible and simple technique. Although some studies have failed to find an association between CDM and functional outcome after thrombolytic therapy [15], other studies have found that the presence of CDM is associated with a better functional outcome after t-PA treatment, even in cases in which t-PA treatment has been conducted beyond the 3-hour therapeutic window [7,16,17]. Therefore, the utility of CDM for the selection of ischemic stroke patients for thrombolytic treatment remains unclear.

Several molecular markers have been identified as diagnostic and prognostic factors in acute ischemic stroke. In our study, we selected molecular markers that are associated with infarcted brain tissue, including IL-6 [18], NSE [19] and MMP-9 [20], as well as molecular markers that are associated with ischemic penumbra, including IL-10, TNF-α and glutamate [21,22]. We found that patients with CDM had higher levels of IL-10, TNF-α and Glu, which indicates more tissue in the ischemic penumbra, as well as lower levels of NSE and MMP-9, which indicates lower levels of infarcted tissue. These findings suggest that CDM is an appropriate marker of at-risk brain tissue, as we have observed previously [8]. In contrast, our results showed that patients with PDM had higher levels of IL-10 only. Therefore, these results suggest that PDM is a worse surrogate than CDM for the detection of at-risk brain tissue.

Only IL-10 levels were independently associated with a good functional outcome in thrombolytic patients with CDM and PDM. Therefore, we conclude that this molecular marker is the best marker to identify at-risk brain tissue. IL-10 is an anti-inflammatory molecule that is secreted mainly by the lymphocytes and monocytes/macrophages to block pro-inflammatory actions [23]. Several studies have shown the neuroprotective effects of the administration of IL-10 in experimental models of cerebral ischemia [24,25]. Furthermore, patients with better clinical outcomes have shown higher IL-10 levels [26]. In our study, we have observed a negative correlation between IL-10 levels and infarct growth, which further suggests a neuroprotective effect of IL-10. Patients with lower IL-10 levels also showed greater infarct growth during the first 72 hours following the onset of the stroke. Our results showed a correlation between glutamate levels and DWI lesion growth, which suggests that this increase in infarct volume is associated with an excitotoxic effect of Glu. However, this association disappears in patients with IL-10 levels ≥30 mg/mL, which suggests a possible neuroprotective effect that blocks these excitotoxic mechanisms. More studies are needed to confirm these results. Another possible explanation is that high levels of IL-10 may be associated with a higher rate of recanalization. However, we cannot confirm this theory with our study because we do not have data on recanalization after rtPA therapy.

A routine test for IL-10 does not exist. Currently, IL-10 levels are determined by commercial ELISA kits. However, this method is simple and inexpensive and could be used in the future within Emergency Departments to facilitate the selection of candidates for recanalization therapies.

Our study has some limitations. First, PWI-MRI was performed in a small percentage of patients. This small sample size may have prevented us from reaching statistical significance to identify the utility of PDM in selecting patients for revascularization techniques. Second, this exploratory study has not prospectively investigated an *a priori* hypothesis. Therefore, these results should be confirmed in future studies. Finally, we did not control for potential confounding variables, such as pre-treatment with statins, which may interfere with molecular marker levels.

In conclusion, IL-10 levels ≥30 mg/mL increase the ability to identify salvageable brain tissue in patients with CDM and PDM and facilitates the selection of ischemic stroke patients for systemic thrombolysis.

## Conclusions

This project has been partially supported by grants from the Spanish Ministry of Science and Innovation (Fondo de Investigaciones Sanitarias, Instituto Salud Carlos III, RETICS-RD06/0026 and PI081472) and Xunta de Galicia (Consellería de Economía e Industria: 09CSA057918PR, Consellería de Sanidade: PS09/32).

P R-C and D B are funded by Instituto de Salud Carlos III (Programa Miguel Servet and Programa Sara Borrell respectively).

## Competing interests

The authors report no conflicts of interests.

## Authors’ contributions

To qualify for authorship, we indicate the contribution of each author to this manuscript: MR-Y, M C, TS, JC and MB have conceived and designed the research; analysed and interpreted the data; and handled funding and supervision. MR-Y, TS and MB have drafted the manuscript and JC has performed the statistical analyses. JS, AD, JC and MB assisted in the analysis and interpretation of the data and contributed critical revisions to the manuscript of important intellectual content. TS and DB acquired, analysed and interpreted the molecular data and supervised the study. PR-C, SP and JAC assisted with the acquisition, analysis and interpretation of the clinical-radiological data and contributed critical revisions to the manuscript of important intellectual content. All authors read and approved the final manuscript.

## Pre-publication history

The pre-publication history for this paper can be accessed here:

http://www.biomedcentral.com/1471-2377/13/62/prepub

## Supplementary Material

Additional file 1: Table: S1 Intra- and inter-assay coefficients of variation (CV) for each molecular marker. Click here for file
