# Supplementary material for: Interleukin-10 facilitates the selection of patients for systemic thrombolysis
Source: BMC Neurol. 2013 Jun 17;13:62. doi: 10.1186/1471-2377-13-62 (PMC3710209; doi:10.1186/1471-2377-13-62)
Supplement: Additional file 1: Table: S1 — Intra- and inter-assay coefficients of variation (CV) for each molecular marker. [file 1471-2377-13-62-S1.doc]

**SUPPLEMENTAL MATERIAL**

**Table e-1.**

Intra- and inter-assay coefficients of variation (CV) for each molecular marker.

|  | **Intra-assay CV (%)** | **Inter-assay CV (%)** |
| --- | --- | --- |
| IL-6 | 3.5 | 5.1 |
| TNF-α | 3.5 | 5.8 |
| NSE | 2.5 | 4.2 |
| IL-10 | 5.1 | 6.7 |
| Active MMP-9 | 3.6 | 6.6 |
| Glutamate | 1.7 | 2.3 |
